# Modulation of Conductivity of Alginate Hydrogels Containing Reduced Graphene Oxide through the Addition of Proteins

**DOI:** 10.3390/pharmaceutics13091473

**Published:** 2021-09-15

**Authors:** Ahmed Raslan, Jesús Ciriza, Ana María Ochoa de Retana, María Luisa Sanjuán, Muhammet S. Toprak, Patricia Galvez-Martin, Laura Saenz-del-Burgo, Jose Luis Pedraz

**Affiliations:** 1 NanoBioCel Group, Laboratory of Pharmacy and Pharmaceutical Technology, Faculty of Pharmacy, University of the Basque Country UPV/EHU, 01006 Vitoria-Gasteiz, Spain; drrayad@gmail.com; 2 Biomedical Research Networking Center in Bioengineering, Biomaterials, and Nanomedicine, CIBER-BBN, Paseo de la Universidad 7, 01006 Vitoria-Gasteiz, Spain; jeciriza@gmail.com; 3 Tissue Microenvironment (TME) Lab, Aragón Institute of Engineering Research (I3A), University of Zaragoza, C/Mariano Esquillor s/n, 50018 Zaragoza, Spain; 4 Institute for Health Research Aragón (IIS Aragón), 50009 Zaragoza, Spain; 5 Department of Organic Chemistry I, Faculty of Pharmacy and Lascaray Research Center, University of the Basque Country (UPV/EHU), Paseo de la Universidad 7, 01006 Vitoria-Gasteiz, Spain; anamaria.ochoaderetana@ehu.eus; 6 Instituto de Ciencia de Materiales de Aragón (Universidad de Zaragoza-CSIC), Facultad de Ciencias, 50009 Zaragoza, Spain; sanjuan@unizar.es; 7 Biomedical and X-ray Physics, Department of Applied Physics, KTH-Royal Institute of Technology, 10691 Stockholm, Sweden; toprak@kth.se; 8 R&D Animal and Human Health, Bioibérica S.A.U., 08029 Barcelona, Spain; pgalvez@bioiberica.com; 9 Bioaraba Health Research Institute, Jose Atxotegi, s/n, 01009 Vitoria-Gasteiz, Spain

**Keywords:** hydrogel, alginate, reduced graphene oxide, conductivity, collagen

## Abstract

Modifying hydrogels in order to enhance their conductivity is an exciting field with applications in cardio and neuro-regenerative medicine. Therefore, we have designed hybrid alginate hydrogels containing uncoated and protein-coated reduced graphene oxide (rGO). We specifically studied the adsorption of three different proteins, BSA, elastin, and collagen, and the outcomes when these protein-coated rGO nanocomposites are embedded within the hydrogels. Our results demonstrate that BSA, elastin, and collagen are adsorbed onto the rGO surface, through a non-spontaneous phenomenon that fits Langmuir and pseudo-second-order adsorption models. Protein-coated rGOs are able to preclude further adsorption of erythropoietin, but not insulin. Collagen showed better adsorption capacity than BSA and elastin due to its hydrophobic nature, although requiring more energy. Moreover, collagen-coated rGO hybrid alginate hydrogels showed an enhancement in conductivity, showing that it could be a promising conductive scaffold for regenerative medicine.

## 1. Introduction

Hydrogels are three-dimensional scaffolds made up of highly hydrophilic polymers. Because they absorb so much water, these hydrogels swell, representing a high degree of flexibility, closer to that of natural tissue [1]. Hydrogels also represent high porosity, excellent biocompatibility, and controllable degradability [2], triggering their application in biomedicine including, applications of soft contact lenses in the correction of vision [3], developing a tissue engineering process [2,4,5], diagnostics [6], and embolizing cells [7]. Depending on the type of bonding, these hydrogels can be classified as either physical or chemical. Physical bonding, such as hydrogen bonding, and hydrophobicity result in physical gels, which are often reversible and affected by environmental factors [1]. Chemical gels, in contrast, are formed by covalent bonding between polymers. These hydrogels are permanent and stable [8,9].

However, there are many limitations to the applications of nature hydrogels in clinical applications. These include high water content, large pores, weak mechanical strength, and fast drug release [3,10].

In the course of time, natural hydrogels have been gradually replaced by synthetic hydrogels that have a longer half lifetime and high mechanical strength [11].

Incorporating a special chemical group into the hydrogel will improve its functionality and allow the hydrogel to be switched by heat, light, magnetic fields, chemical agents, or pH alterations [2,12,13]. Functionalized hydrogels with therapeutic peptides and proteins are also possible. These can be used to treat diseases, such as cancer, immune disorders, mental disorders, hypertension, and certain cardiovascular and metabolic problems.

Extracellular matrix (ECM) is a non-cellular component of tissue that provides physical support to cells. Emerging research has shown that ECM provides tissue-specific biochemical and biophysical cues required for tissue morphogenesis [14].

For decades, alginate was considered one of the best biomaterials for assembling and fabricating functional hydrogels, owing to its excellent biocompatibility and high porosity [15,16]. However, several drawbacks, such as its mechanical strength, weakness, the leak of cell adhesion, and its rapid drug release, have limited its clinical application [17,18]. To solve these drawbacks, different materials have been integrated into the alginate matrix, also creating biomimetic support. In this regard, graphene has been applied in various fields based on this excellent characteristic, including electronics [19], being considered a strong candidate in the field of biomedicine, both for fabricating drug delivery vehicles and gene therapy [19,20,21,22]. However, studies with graphene are contradictory [22]. On one hand, some reports describe graphene as a material that does not cause any alteration in cell function [23,24], with acceptable hemocompatibility, and without induction of immune response, even at high concentrations [24]. On the other hand, reports show a cytotoxic effect even at a low dosage [23,25]. Graphene oxide (GO), a derivative from graphene, can be produced through Hummer’s method [26,27,28,29,30] and shows unique physical and mechanical properties, including high thermal conductivity [26,31,32], colossal surface area [33,34], and a robust mechanical strength [35,36]. The oxidation process of graphene alters the surface of graphene, increasing its affinity to water [37,38] and, therefore, mediating a vast number of biochemical reactions and bio-conjugations along its surface [39]. GO biocompatibility is affected in two-dimensional cultures by factors such as GO surface processing and the particle size of surface functionality, with impact on adhesion or cell proliferation [40]. In this regard, our group combined GO with alginate to modify alginate surface properties and its mechanical strength, showing good biocompatibility with myoblasts in alginate microcapsules, within a range of GO concentrations. Concisely, GO concentrations between 25 and 50 µg/mL enhanced the viability of C_2_C_12_ myoblasts [16,40,41,42]. However, the integration of GO within alginate matrices reduced the release of therapeutic factors, since GO could sorb the secreted therapeutic factors on its surface due to its high surface activity. GO sorption was solved by applying a pre-coating layer on the GO surface with fetal bovine serum [16,40,41,42].

An alternative graphene derivate is reduced GO, with low surface absorbability compared to GO [43]. Several techniques have been utilized to reduce GO, including mechanical reduction or chemical reduction [43], adding alterations in rGO surface, such as the chemical structure and hydrophilicity [37,44]. However, there is again conflicting information comparing the biocompatibility of GO and rGO [43,45,46]. It has been described that the irradiated light reduction in GO yields an immense reactive oxygen species generation and oxidative stress [43], while the small particle size of thermally reduced oxide can stimulate cytotoxicity, facilitating its cell membrane penetration [43]. However, chemically reduced rGO shows lower toxicity than other rGO forms [47]. Modified scaffolds with rGO have shown strong mechanical strength and ultra-high electrical conductivity [48], with favorable impacts on cell viability, proliferation, and differentiation [48]. Hydrothermal processing of alginate and graphene oxide in an aqueous solution yields hybrid alginate-rGO hydrogels with high porosity. In the hydrothermal process, graphene nanosheets and alginate form a porous structure as a result of auto-assembly; afterwards, the hybrid hydrogel is produced by ionically linking polymer networks of alginate [49]. Thus, rGO has become more applicable in tissue engineering, particularly for neuronal regeneration [48,50] or cardiomyocytes regeneration [51]. Here, we are using reduced graphene-based materials, as it is one of the best redox species that could be studied on an electrode. The redox peaks will give a clear indication of changes to the double layer on the electrode’s surface. When rGO-protein-alginate is incorporated over an electrode the double layer changes, this can affect the double-layer capacitance and the electron transfer resistance. Therefore, by monitoring the charge transfer resistance, we could understand the charge transfer properties of the double layer. Cyclic voltammetry (CV) and electrochemical impedance spectroscopy (EIS) are interrelated electroanalytical methods to examine the electrochemical double layer on the electrodes. In this work, we aimed to create conductive protein-rGO-alginate hydrogels using different proteins in order to study their adsorption capacity and electrochemical characteristics to identify the best composition.

## 2. Materials and Methods

### 2.1. Materials

The chemically reduced graphene oxide (rGO) powder was provided by Graphenea (San Sebastian, Spain). Bovine Serum Albumin (BSA) and type 1 Collagen were purchased from Sigma Aldrich (Saint Louis, MO, USA). Elastin was provided by Bioiberica (Barcelona, Spain). High pure, low-viscosity, and ultra guluronic (LVG) acid alginate was purchased from FMC Biopolymer (Drammen, Norway).

### 2.2. Protein Adsorption

rGO powder in a 3/1 mixture of water and DMSO (*v*/*v*) to get a suspension of 4 mg/mL rGO, homogenizing by sonication for 60 min in a bath. The resulting rGO dispersion was diluted to 2.5 mg/mL with 18 MΩ cm resistivity deionize water (DI). Then, 90 μL of 200 μg/mL BSA, 200 μg/mL elastin, or 500 μg/mL collagen were mixed with 10 μL of 250 μg/mL rGO suspension for 120 min at 37 °C under agitation at 400 rpm. The samples were spun down at 15,000 rpm for 15 min to collect supernatants. The lack of adsorbed protein was determined with the BCA kit (Thermo Fisher, Massachusetts, MA, USA) in a M 200 TECAN microplate reader (TECAN Trading AG, Männedorf, Switzerland) at 562 nm. At least three samples were quantified to ensure accuracy and repeatability. The % of protein sorption was estimated by Equation (1), and the adsorption capacity qe (µg/µg) was estimated by Equation (2), where C_0_ (µg/mL) and Ce (µg/mL) are the original protein concentration and the protein concentration at steadiness, respectively, S is the sample volume (mL), and m is the mass of rGO (µg).

Adsorbability (%) = (C_0_ − Ce) × 100/C_0_ (1)

Adsorption capacity qe = (C_0_ − Ce) × S/m (2)

Freundlich and Langmuir’s adsorption isotherm models were implemented to estimate the adsorption isotherm. The Langmuir model is displayed in Equations (3) and (4), where the concentration of the adsorbed protein at steady-state is Ce (µg/mL), qe is the adsorption capacity (µg/ug), qmax (µg/µg) is the maximum quantity of protein sorbed per unit mass of rGO, K_L_ (mL/µg) is the Langmuir factor associated with the surface affinity for the protein, C_0_ is the initial protein concentration, and R_L_ is the separation factor that specifies the Langmuir isotherm’s fundamental aspects [52].

Ce/qe = Ce/qmax + 1/(qmax·K_L_) (3)

R_L_ = 1/ (1 + K_L_·C_0_) (4)

Freundlich model is described as follows (Equation (5)), where K_F_ and m are the Freundlich constant and intensity adsorption, respectively:

Log qe = log K_F_ + 1/m·log Ce (5)

### 2.3. Kinetics of Protein Adsorption

250 µg/mL rGO was suspended either in 50 µg/mL BSA, 200 µg/mL collagen, or 50 µg/mL elastin by agitation at 37 °C. After spinning down at 15,000 rpm for 10 min, supernatants were collected after the following incubation times: 5, 10, 20, 30, and 80 min. Adsorbed protein was quantified with a BCA kit (Thermo Fisher) in a M 200 TECAN microplate reader (TECAN Trading AG, Männedorf, Switzerland) at 562 nm. At least three samples were quantified to ensure accuracy and repeatability. Intra-particle diffusion model, pseudo-first-order, and pseudo-second-order rate adsorption were applied to the results to determine the most appropriate adsorption kinetic model. At least three samples were quantified for each condition. Several models were evaluated to elucidate the adsorption mechanism and the adsorption rate (Equations (6)–(9)) [16], where qt (µg/µg) is the quantity of the adsorbed protein vs. time (*t*) (min.), Kp is the intra-particle diffusion rate constant (µg/µg. min^1/2^), and C (µg/µg) is a constant for the intra-particle diffusion model, which provides a piece of information about the thickness of the barrier layer [52,53], qe (µg/µg) is the adsorption capacity at equilibrium, K_1_ (min^−1^) is the constant rate of the pseudo-first-order model, and K_2_ (µg·µg^−1^min^−1^) is the pseudo-second-order model’s constant rate.

q*t* = Kp.*t*^1/2^ + C (6)

log (qe − q*t*) = log (qe) − (K_1_·*t*)/2.303 (7)

*t*/q*t* = *t*/qe + 1/K_2_·1/(qe)^2^ (8)

1/q*t* = 1/(K_2_ + qe^2^)·1/*t* + 1/qe (9)

### 2.4. Thermodynamics of Protein Adsorption

250 µg/mL rGO was suspended either in 50 µg/mL BSA, 200 µg/mL collagen, or 50 µg/mL elastin solution. Mixtures with each protein were incubated and agitated at the following temperatures: 5, 10, 15, 25, 37, and 39 °C. After spinning down at 15,000 rpm for ten minutes, supernatants were collected to quantify the amount of non-adsorbed protein with BCA kit (Thermo Fisher) in a M 200 TECAN microplate reader (TECAN Trading AG, Männedorf, Switzerland) at 562 nm. At least three samples were quantified for each condition. The fundamental thermodynamic factors, such as entropy change (∆S°), enthalpy change (∆H°), and Gibbs free energy change (∆G°), were calculated using Equations (10)–(13) [53,54,55], where R is the gas constant (8.314 J/mol K), T the absolute temperature (K), K_d_ the equilibrium constant, qe (µg/µg) the quantity of protein adsorbed per mass unit of rGO at equilibrium, and Ce (µg/mL) the equilibrium concentration of each protein:

Kd = qe/Ce(10)

∆G° = −R·T·lnKd(11)

lnK_d_ = −∆H/R·T + ∆S/R (12)

∆G° = ∆H − T∆S(13)

### 2.5. Characterization of the rGO-Protein Binding

The binding of each protein to rGO surface was studied by Raman and Fourier transform infrared spectroscopy. First, 250 µg/mL rGO was suspended either in 50 µg/mL BSA, 200 µg/mL collagen, or 50 µg/mL elastin solution, and agitated at 37 °C for 2 h. The supernatants were collected after spinning down at rpm for 15 min and lyophilized in a Telstar Lyobeta 15 lyophilizer. rGO without protein incubation and proteins without rGO were also studied. At least three samples were studied for each condition.

Raman spectrum was obtained by confocal Raman imaging (Alpha 300 M, Company WITec, Ulm, Germany) with a 532 nm laser (5% laser power, a contact time of the 50 s, and four accumulations). FT-IR, using an attenuated total reflectance (ATR) technique, was performed in a FT-IR Bruker IFS 66/S Spectrometer, with 32 scans at a resolution of 4 cm^−1^ between the wavelength ranges of 4000–400 cm^−1^. Air background was applied as a blank. At least three samples were analyzed for each condition.

### 2.6. EPO and Insulin Adsorption Blocking Study

100 µg/mL rGO was suspended either in DI water, 50 µg/mL BSA, 200 µg/mL collagen, or elastin and incubated for two hours at 37 °C. After being spun down at 15,000 rpm for 15 min, supernatants were removed, and protein-coated rGO was incubated with 200 µL of 200 mIU/mL recombinant EPO or 150 mIU/mL recombinant insulin for 24 h at 37 °C. Uncoated rGO was used as a reference. Next, samples were spun down by centrifuging for 15 min at 15,000 rpm and supernatants were collected. Non adsorbed EPO and insulin were quantified with Quantikine IVD EPO (R&D Systems) and Insulin Elisa (Mercodia), respectively, following manufacturer recommendation. At least three samples were analyzed for each condition.

### 2.7. Preparation of Alginate Hydrogels Containing rGO and Protein-Coated rGO

At room temperature, 1.87 g of high pure sodium alginate was dissolved in 1% mannitol by magnetic string at 200 rpm for 2 h; then, it was mixed and homogenized with either rGO or protein-coated rGO suspension, obtaining a final concentration of 1.5% alginate and 50 μg/mL rGO. To prepare hybrid alginate hydrogels, alginate solutions were mixed with 60 μL of 1.22 M calcium sulfate through a connector (Braun) between two Luer Lock syringes (BS Syringe). The mixed solutions were dispensed between two glass slides with 2 mm spacing, leaving them for 30 min to form hydrogel disks, 14 mm in diameter.

### 2.8. Conductivity of Alginate Hydrogels Containing Protein-Coated rGO

Electrochemical impedance spectra (EIS) were measured using a potentiostat (Princeton Applied research, Oak Ridge, TN, USA), with a screen-printed electrode (Dropsens, Oviedo, Spain) based on carbon, and a silver electrode as reference. Samples were immersed in 0.1 M PBS buffer at room temperature, applying frequency series from 10^−1^ to 105 Hz. Cyclic Voltammetry (CV) measurements were performed in 0.1 M PBS buffer between the potential range from −0.2 to 0.5 V and at different scan speeds (100 mVs^−1^). Specific capacitance was estimated from CV curves by Equation (14) [16,56], where C (F·g^−1^) is the specific capacitance, Q the mean charge throughout the charging and discharging procedure, the potential range V (Volt), and the mass m (g) of the hydrogel disk:

C = Q/(2Vm)(14)

### 2.9. Statistical Analysis

Statistical analysis was performed with GraphPad Prism 9.0 (GraphPad Inc., San Diego, CA, USA) and SPSS (version 27.00, IBM, New York, NY, USA) software. Results were presented as mean ± standard deviation. A normality test was performed, considering *p* < 0.05 as statistically significant values after ANOVA and Tukey’s post hoc test bivariate correlation testing.

## 3. Results and Discussion

### 3.1. Adsorption of Proteins on rGO Surface

**Figure 1:**
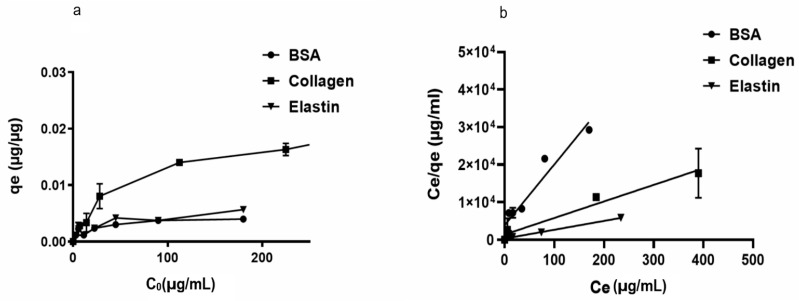
(**a**). Adsorption capacity (qe) of rGO (250 µg/mL) with initial concentrations of 200 μg/mL BSA, 200 μg/mL elastin and 500 μg/mL collagen after two hours of incubation at 37 °C. (**b**) Langmuir models for BSA, collagen, and BSA adsorption on the rGO surface.

We began studying the adsorption capacity of rGO to several proteins usually located in FBS. Thus, we observed that rGO adsorption capacity (qe) was enhanced at low protein dose values, indicating the presence of available active groups on the rGO surface. However, no significant modifications in qe values at high initial protein doses (Co) were quantified, suggesting that no further protein loading on the rGO surface was allowed. Among the studied proteins, collagen showed the highest adsorption capacity (qe = 0.022 µg/µg), while other hydrophilic proteins, such as BSA and elastin showed a low affinity for rGO surface (qe between 0.0049–0.0067 µg/µg) (Figure 1a).

In order to understand the adsorption phenomena involved on rGO surface, Langmuir and Freundlich’s models were applied to the qe values recorded at a constant temperature, calculating the required parameters for the aforementioned models (Table 1 and Figure 1b). Protein adsorption phenomena on the rGO surface was better specified by the Langmuir than the Freundlich model, with R2 values between 0.968 and 0.996 for the Langmuir model and convergence between calculated qmax values and experimental qe results. Therefore, we suggest that adsorption phenomena occur on a homogeneous surface of rGO, with a specific number of adsorption sites on rGO surface binding to protein active sites and forming a monolayer [56]. In fact, Langmuir variable values (RL) < 1 would suggest advantageous adsorption onto the rGO surface for the studied proteins, with irreversibile adsorption for collagen and elastin (RL ≈ 0) [57].

**Figure 2:**
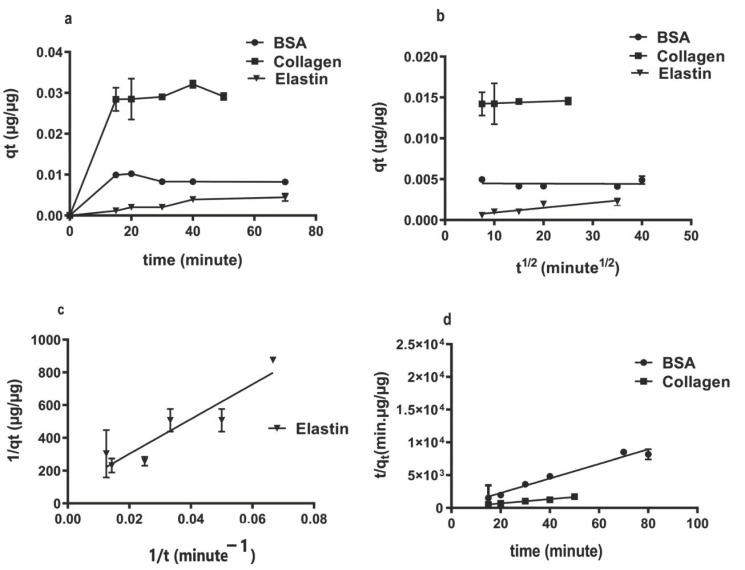
Kinetic protein adsorption models on rGO surface. (**a**) Adsorption capacity over time; (**b**) intra-particle diffusion model plot; (**c**) pseudo-second-order model plot for BSA and collagen; (**d**) nonlinear plot of pseudo-second-order for elastin.

Studying the adsorption capacity (qt) of rGO over time, we observed a quick adsorption process, completed after 20 min (Figure 2a). The intraparticle-diffusion model was implemented to attain a suitable mechanism that fits the protein adsorption on rGO surface (Figure 2b). However, since q*t* vs. *t*_1/2_ plotting showed linearity without going across zero, we think that intraparticle-diffusion is not the only process controlling protein adsorption on the rGO surface; film diffusion also contributes to the protein adsorption [40]. Calculated intraparticle-diffusion parameters, such as the diffusion rate constant (KP) and the impediment layer wideness (C) (Table 2), showed collagen with the highest intra-particle diffusion rate Kp (0.0001 µg·µg^−1^min^−1/2^) and boundary layer thickness (0.0132), correlating to a strong hydrophobic attraction between collagen and the hydrophobic rGO surface. This result suggests that π–π bonding between collagen and rGO might also be responsible for this bonding [57]. However, the hydrophilicity of BSA and elastin would result in the decrease in interference forces and therefore the affinity with rGO.

Among adsorption kinetic mathematical models, the low R^2^ values calculated in the pseudo-first-order model (0.35–0.82), with vast difference between estimated qe and experimental qt (Table 2), discarded this model for describing adsorption phenomena on the rGO surface. However, the pseudo-second-order model showed high R^2^ values (0.93–0.99) and convergence between the estimated qe and the experimental qt values (Table 2); therefore, this model can be considered the best kinetic model to define the studied protein adsorption phenomena on the rGO surface [58,59]. Interestingly, pseudo-second-order constant K_2_ reduced while hydrophilicity increased with the lowest K_2_ for elastin (2.16·µg^−1^·in^−1^) and with the highest for collagen (27.27 µg·µg^−1^·min^−1^). Representing qt values versus time, while a linear pseudo-second-order plot was more suitable for collagen and BSA (Figure 2c), a nonlinear pseudo-second-order plot was fit for elastin adsorption (Figure 2d) [60]. Collagen showed a ten-fold higher adsorption rate constant (K_2_) than BSA and elastin, indicating the highest affinity for rGO than elastin or BSA and due to the strong hydrophobic–hydrophobic interactions between rGO surface and collagen [61].

### 3.2. Thermodynamics of Protein Adsorption onto rGO Surface

**Figure 3:**
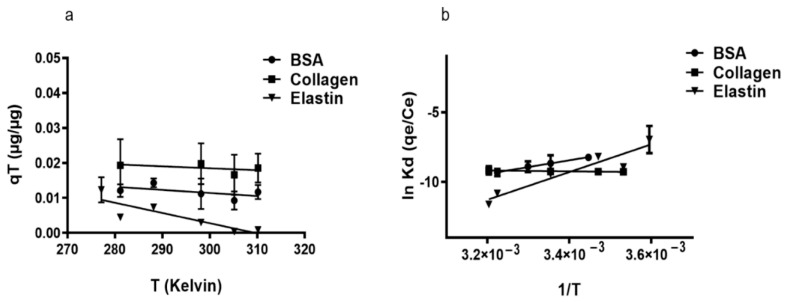
(**a**) Influence of temperature on the adsorption of proteins. Where mixtures of 250 µg/mL rGO were suspended either in 50 µg/mL BSA, 200 µg/mL collagen, or 50 µg/mL elastin solution and incubated for 2 h at the following temperatures: 5, 10, 15, 25, 37, and 39 °C. (**b**) Van’t Hoff linear plot of ln Kd against 1/T for proteins adsorption on rGO. ΔH° was estimated from the slope (= −ΔH°/R) and ΔS° from the y-intercept (= +ΔS°/R).

The study of adsorption capacity (q_T_) with BSA and elastin on rGO surface increasing temperature revealed the exothermic character (∆H° < 0) of the adsorption (Figure 3a, Table 3). However, collagen showed endothermic adsorption when the temperature was increased (∆H° = 2.44 kJ/mol). We consider that the protein adsorption could start with an endothermic hydration step, followed by exothermic adsorption, but in collagen, there would be a hydration step caused by its hydrophobic nature that requires more energy than the other studied proteins [62]. This hypothesis would explain why an increase in the temperature would enhance the adsorption capacity of collagen, while it would increase the kinetic energy of BSA and elastin causing their elution from rGO surface. Moreover, the low ΔG° values (Table 3) indicated the physio-sorption nature of the adsorption [58], a non-spontaneous phenomenon that is a feature of positive ΔG° values. It could be attributed to the presence of an energy barrier in the migration of the studied proteins towards the rGO surface, with water forming a hydration shell around the proteins that would hinder their adsorption on rGO. Finally, the remarkable reduction in entropy during the adsorption of the studied proteins [58] suggests that molecular motion at the solid–liquid interface is more organized [63].

### 3.3. Surface Chemistry of Protein Adsorbed rGO

**Figure 4:**
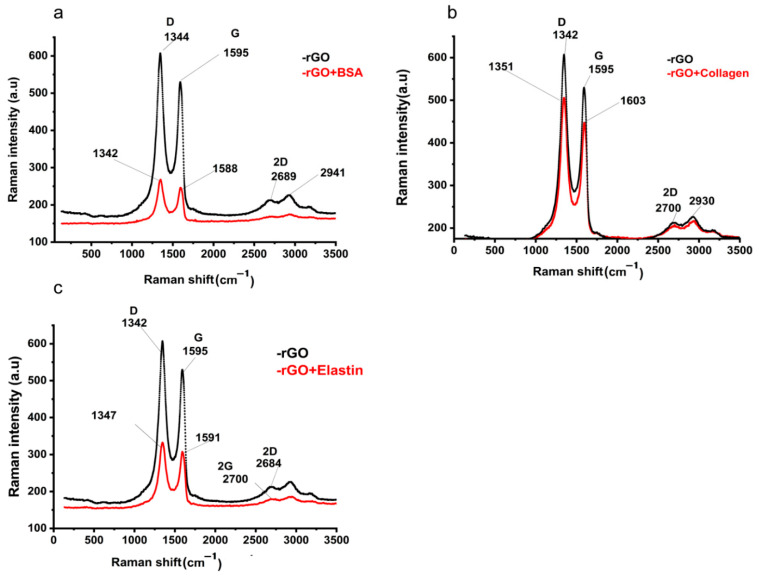
Raman spectra of rGO before and after the adsorption of (**a**) BSA (rGO + BSA), (**b**) collagen (rGO + collagen), and (**c**) elastin (rGO + elastin).

In order to confirm the adsorption of the studied proteins onto the rGO surface, we studied rGO and protein-adsorbed rGO by Raman spectroscopy and FT-IR. In Raman spectra, G and D bands at ~1595 cm^−1^ and ~342 cm^−1^ in rGO Raman spectrum indicated the occurrence of defects due to the reduction process of GO [52] (Figure 4a), also detected in protein adsorbed-rGO samples (Figure 4b–d). After proteins were adsorbed on the rGO surface, the G and D bands shifted to ~1588–1601 cm^−1^ and ~1342–1351 cm^−1^, respectively. The intensity ratio between those bands (ID/IG) suggested an sp^2^ electron distribution in all the samples [63], being higher than those ID/IG ratios previously described in graphene [64], and slightly increased when proteins were adsorbed on rGO (Table 4). Finally, the 2D band position and their intensity ratio with G band (2D/G) increase would indicate more structural defects, most likely attributed to protein adsorption on rGO [65], confirming the adsorption of the studied proteins on the rGO surface [66].

**Figure 5:**
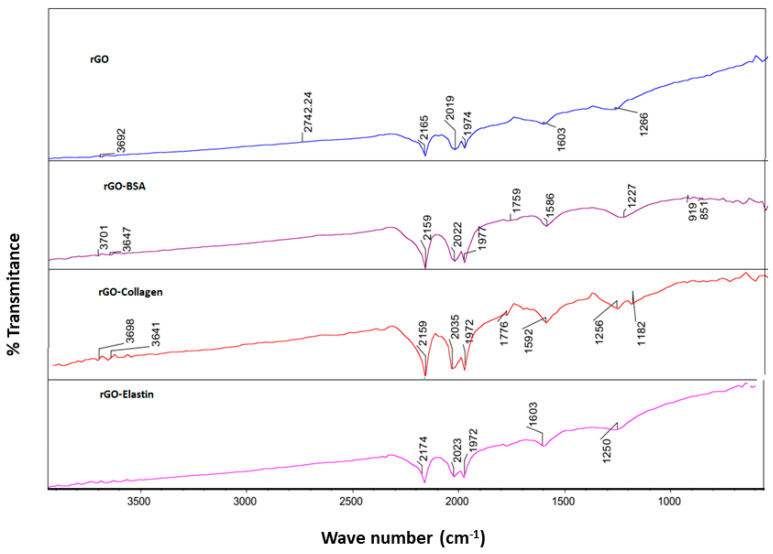
FT-IR spectra of rGO before and after the adsorption of BSA (rGO-BSA), collagen (rGO-collagen), and elastin (rGO-elastin).

Then, we confirmed the adsorption of the studied proteins on the rGO surface by FT-IR using an attenuated total reflectance (ATR) technique. Thus, we detected an absorption peak in rGO sample at 1603 cm^−1^ related to C=C stretching vibration, and at 1736 cm^−1^ and 1266 cm^−1^, corresponding to carboxyl C=O and carbonyl C-O stretching vibrations, respectively [56] (Figure 5). When rGO incubated with the studied proteins was analyzed by FT-IR, an obvious shift of the C=C stretching band from 1603 cm^−1^ to 1590–1600 cm^−1^ and a shift of the C-O stretching band from 1266 cm^−1^ to 1227–1250 cm^−1^ were detected, which were ascribed to the π–π bonding between the benzene ring from proteins and the rGO surface (Table 5) [67]. Although due to the incomplete reduction in rGO, a hydroxyl signal was detected in FT-IR spectra, forming H-bonding when incubated with proteins (3692–3701 cm^−1^), these slight changes are not strong enough for stable H-bond formation. Therefore, we consider that the studied protein adsorption on the rGO surface is attributed mainly to π–π interactions between the rGO surface and the proteins [52,68].

### 3.4. Therapeutic Protein Adsorption on Protein Coated rGO

**Figure 6:**
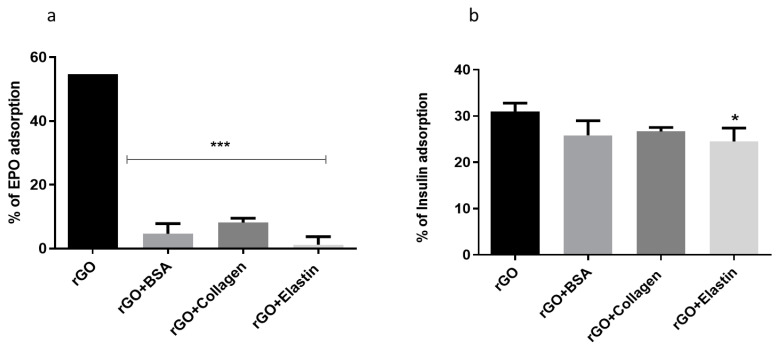
(**a**) Erythropoietin (EPO) and (**b**) insulin adsorption on rGO and rGO-protein matrix. Note: *: *p* < 0.05, ***: *p* < 0.001, compared to rGO without protein coating.

In order to determine if blocking the rGO surface with the studied proteins precluded further adsorption of other proteins, we studied the adsorption of two different therapeutic proteins as erythropoietin and insulin (Figure 6) for 24 h at 37 °C. No differences were detected among the studied coating proteins when adsorption studies were performed with either erythropoietin (EPO) or insulin. However, this behavior with the therapeutic proteins was slightly different, since EPO trapping was lower than insulin, indicating a higher blocking for EPO protein by the protein coating.

The mechanism of insulin adsorption by graphene and GO was shown to be different. The high adsorption of insulin hormone by graphene can be explained by a strong π–π interaction between the phenyl rings of insulin and the graphene surfaces [69]. On the other hand, insulin adsorption on the GO surface was attributed to electrostatic interactions and hydrogen bonds with the oxygenated functional groups. This interaction would be enough to overcome the blocking exerted by the coated studied proteins onto rGO, being unable to block the adsorption of further proteins with low molecular weight (6 kDa) and high hydrophilicity, such as insulin [16].

### 3.5. Conductivity Studies of Alginate Hydrogels Containing Protein Coated rGO

**Figure 7:**
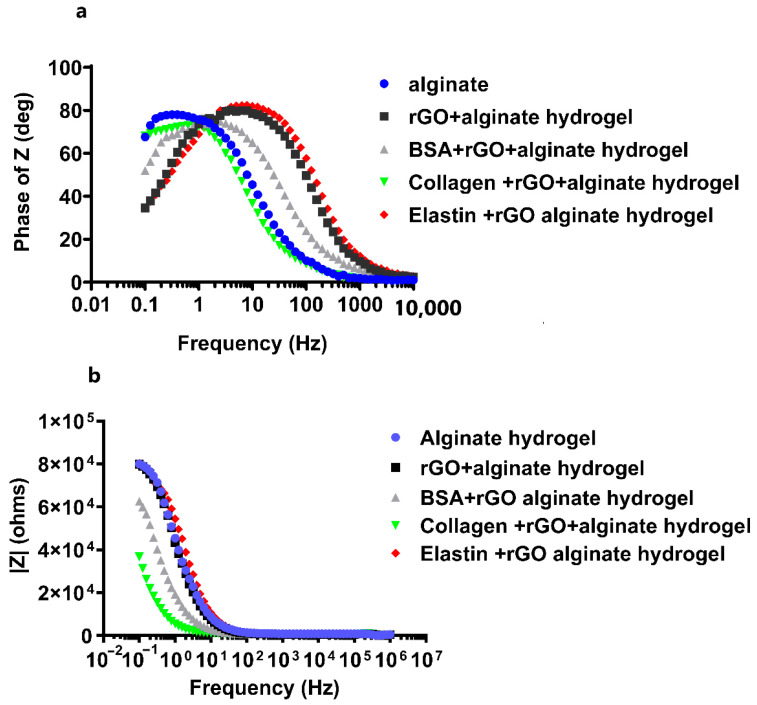
Electrochemical impedance spectroscopy (EIS) measurements for pristine alginate hydrogel, and hybrid alginate hydrogels containing protein-coated rGOs (rGO-BSA, rGO-Collagen, and rGO-Elastin). Bode plots representing (**a**) phase angle Z vs. frequency, and (**b**) the impedance modulus |Z| (ohms) vs. frequency. Measurements were performed in 0.1 M PBS buffer at room temperature.

rGO has properties close to graphene and shows excellent electrical properties, including high electrical conductivity and high mobility [43]. During the reduction process, structural defects are formed, such as physical holes after removing the oxygen functional groups or alkyl groups. These holes can act as charge carriers and are responsible for the high conductivity of rGO [56]. In fact, the incorporation of rGO within scaffold matrices has improved their conductivity, such as rGO–gelatin methacryloyl (GelMA) hybrid hydrogels displaying improved electrical conductivity and mechanical properties [63]. Hybrid scaffolds with carbon nanofibers (CNFs) incorporated in alginate and gelatin hydrogels have demonstrated high electroconduction. Furthermore, this preparation method permits the elaboration of homogeneously dispersed hydrogels with integrated CNFs. The hybrid composite hydrogels including rGO were reported to display excellent electrical conductivity in the range of 4.1 × 10^−4^ ± 2 × 10^−5^ S/cm [70]. Therefore, we decided to study the electrochemical activity of protein-coated-rGO embedded within alginate hydrogels through the impedance spectroscopy (EIS) and cyclic voltammetry (CV) measurements. We quantified the phase angle (deg) and impedance modulus |Z| (ohms) within a range of frequencies between 10^−1^ and 10^6^ Hz in EIS, followed by analyzing the experimental data by Bode model (Figure 7) to determine the insulating or conducting behavior for the studied hydrogels. We determined that the addition of rGO or protein-coated-rGO to alginate matrix induced a slight decrease in hydrogel impedance at low frequencies. Moreover, we observed that the phase angle stayed close to 90° at low frequencies, decreasing towards zero at ultra-high frequencies in all the hydrogels studied (Figure 7a), quantifying the highest phase angle in collagen-coated rGO-alginate hydrogels. The magnitude of the impedance is inversely proportional to capacitance: ideal capacitors have lower impedance [71]. With rising frequency, the impedance of any given capacitance decreases. The frequency response of impedance is depicted in two portions on the Bode plot (Figure 7a). The first is associated with the hydrogel and is below 10^1^ Hz, whereas the second is related to the charge transfer resistance between the hybrid hydrogel and the electrolyte [72].

**Figure 8:**
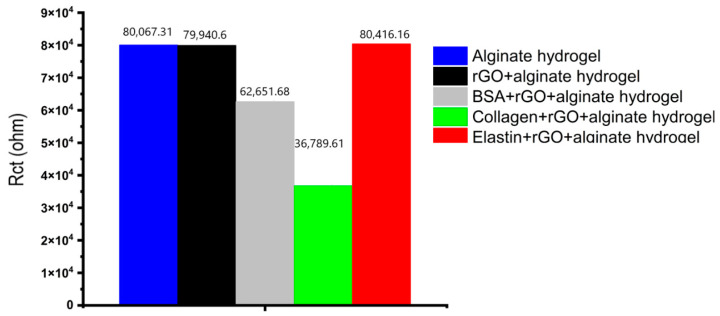
The charge transfer resistance (Rct) derived by analyzing the EIS spectra.

The charge transfer resistance, as seen in Figure 7b, has the largest variance, where alginate hydrogels showed the highest impedance value, decreasing when rGO or protein-rGO was introduced into the hydrogels, especially with collagen that showed the lowest impedance value. At low frequencies, the charge transfer resistance can be obtained by interpolating the semi-circle to the real x-axis [73]. The current density of the rGO-proteins alginate hydrogels rises as the hydrogel resistance and charge transfer resistance fall [73]. Figure 8 depicts the charge transfer resistance (Rct) derived by analyzing the EIS spectra. Because the charge transfer resistance characterizes the electron flow at the counter electrode to a great extent, the lower the resistance, the faster the electron flow rate in the hydrogel [73].

The impedance of rGO-proteins alginate hydrogels reduced greater and that was attributed to the π–π bonding between rGO and the adsorbed proteins [74], the electrons of the π–π bonding have higher mobility than that of the holes in rGO, which is the main charge carrier in rGO [75,76].

**Figure 9:**
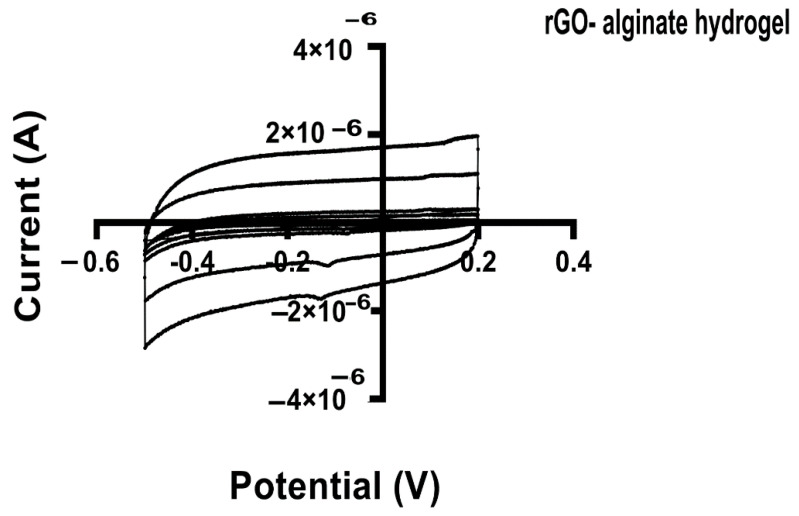
Cyclic voltammograms (CV) for hybrid rGO-alginate hydrogel at various scan rates. The potential (V) was plotted against the current (A). All specimens were dipped in 0.1M PBS and CV measurements were done at the potential range from −0.2 to 0.5 V at different scan rates (100 mVs^−1^).

Cyclic voltammetry (CV) is a technique used to assess redox properties, stability, and surface area of electrodes for biosensing, using materials such as graphene [77]. In this work, we applied the CV to study the electrochemical activity of the alginate hydrogel and hybrid alginate hydrogel with rGO and rGO-proteins. Through the CV the specific capacitance of each hydrogel can be estimated. Voltammetry (CV) measurements showed excellent capacitance for all the tested hydrogels, maintaining the box-like shape even at different scan rates (Figure 9).

**Figure 10:**
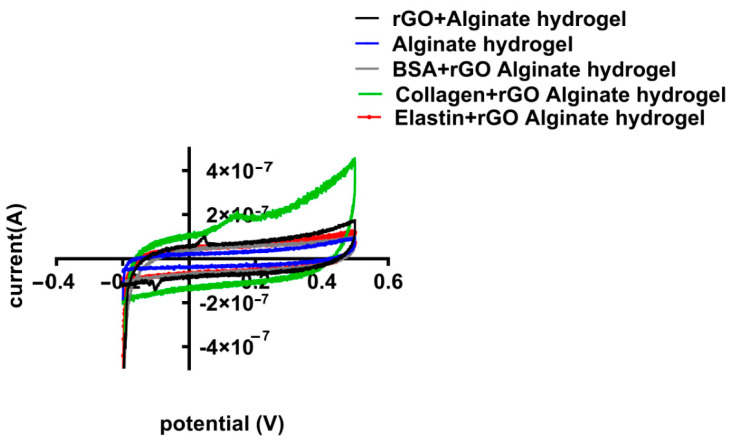
Cyclic voltammetry (CV) potential (V) vs. current curves for different hybrid alginate hydrogels containing uncoated and protein-coated rGOs (rGO-BSA, rGO-Collagen, and rGO-Elastin). All specimens were dipped in 0.1M PBS and CV measurements have been done at the potential range of −0.2 to 0.5 V, though the rate is specified as 100 mV/s.

When rGO-proteins were added to the alginate matrix, the specific capacitance of the hybrid alginate hydrogel was modified (Figure 10). Collagen-coated rGO maximized the capacitance reaching the highest value (3.17 × 10^−5^ F/g), while BSA-rGO-alginate hydrogel and elastin-rGO-alginate hydrogel minimized it (to 1.88 × 10^−5^ F/g and 1.20 × 10^−5^ F/g, respectively) (Table 6). Therefore, CV results confirmed the measurement quantified in EIS, indicating that collagen-coated rGO hydrogel enhances the conducting behavior of rGO hydrogels [74], modifying the electrical properties of alginate hydrogel.

## 4. Conclusions

Aiming to improve alginate hydrogels, we incorporated reduced graphene oxide (rGO) coated with proteins—BSA, collagen, or elastin—into the alginate hydrogel matrix. Our finding demonstrated that the adsorption of these three proteins onto the rGO surface occurs through the π–π interactions. Moreover, the hydrophobic nature of the rGO surface has increased its affinity for hydrophobic protein and decreased its affinity for hydrophilic proteins. Among the studied proteins, the rGO surface showed the highest adsorption capacity to collagen (qe = 0.0220 µg/µg), while BSA and elastin represented three to five times lower qe values (0.0070 µg/µg and 0.0049 µg/µg, respectively). The thermodynamic study showed that the adsorption of these proteins onto rGO is a non-spontaneous phenomenon. Moreover, the adsorption of collagen by rGO is an endothermic process, while the adsorption of BSA and elastin is exothermic. When rGO-protein matrices were incorporated into the alginate hydrogels, the protein coat on the rGO surface was able to preclude further adsorption of erythropoietin. This collagen-coated rGO addition to alginate hydrogel enhanced alginate’s conductivity, leading to the lowest impedance modulus and the highest specific capacitance, 3.17 × 10^−5^ (F/g). This enhancement would be extremely helpful for its application in tissue engineering for neuronal or cardiomyocyte regeneration, cell-based therapy, and tissue engineering.

## Figures and Tables

**Table 1:** Parameters required for Langmuir and Freundlich adsorption isotherms. qe (µg/µg) is the Adsorption capacity at equilibrium, KL (mL/µg) is the Langmuir factor, R2 is the coefficient of determination for the Langmuir model, and qmax (µg/µg) is the maximum adsorption capacity of the proteins by rGO. For the Freundlich model, where KF is the Freundlich constant and m is the intensity adsorption, R2 is the coefficient of determination for the Freundlich isotherm.

Langmuir Model						Freundlich Model			
	q_e_ µg/µg	K_L_ ml/µg	R_L_	R^2^	qmax µg/µg	1/m	m	K_f_	R^2^
BSA	0.0070	0.0297	0.1570	0.968	0.0091	0.345	2.896	0.00197	0.95
Collagen	0.0220	0.0313	0.0660	0.987	0.0230	0.386	2.590	0.00228	0.92
Elastin	0.0049	0.1980	0.0271	0.996	0.0049	0.161	6.184	0.00230	0.83

**Table 2:** Calculated parameters from intra-particle diffusion pseudo-first-order and pseudo-second-order model. Calculated parameters from intra-particle diffusion are the intra-particle diffusion rate constant (K_p_), constant for the intra-particle diffusion model (C) and coefficient of determination (R^2^). The presented parameters for the pseudo-first-order are the adsorption capacity at equilibrium (qe), rate constant (K_1_), and the coefficient of determination (R^2^). For the pseudo-second-order model, the parameters are the rate constant (K_2_) and the adsorption capacity at different times (q*t*).

	Intra-Particle-Diffusion Model			Pseudo First Order Model			Pseudo Second Order Model			
	K_p_ µg·µg^−1^min^−1/2^	C	R^2^	K_1_ min^−1^	qe µg/µg	R^2^	qe µg/µg	K_2_ µg·µg^−1^min^−1^	R^2^	q*t* µg/µg
BSA	0.00002	0.0037	0.67	22.1	0.0074	0.35	0.009	2.34	0.98	0.0091
Collagen	0.0001	0.0132	0.96	16.3	0.0019	0.65	0.0307	27.27	0.99	0.0294
Elastin	0.00005	0.0004	0.91	67.7	0.0379	0.82	0.0067	2.16	0.93	0.0044

**Table 3:** Thermodynamic parameters for protein adsorption by rGO: enthalpy of adsorption, ΔH° (kJ/mol); entropy of adsorption, ΔS° (kJ/mol·K); Gibbs free energy of adsorption, ΔG° (kJ/mol); coefficient of determination, R^2^.

	ΔH° kJ/mol	ΔS° kJ/mol.K	ΔG° kJ/mol	R^2^
BSA	−40.34	−0.207	23.95	0.99
Collagen	2.44	−0.069	23.68	0.95
Elastin	−89.96	−0.358	28.40	0.94

**Table 4:** Parameters from Raman spectra: the D band is related to scattering from local defects or disorders present in carbon, and the G bands arise from the in-plane diverging stretching of the C–C bonds in the graphitic structure, and (2D) is related to the number of graphene layers. ID/IG is the intensity ratio of the D and G bands, which refers to the amount of defects existing in the graphene matter. The intensity ratio of 2D/G is related to the number of graphene layers in the matrix.

Sample	D cm^−1^	G cm^−1^	2D	ID/IG	2D/G
rGO	1342	1595	2664	1.17	0.142
rGO-BSA	1342	1588	2669	1.19	0.226
rGO-Collagen	1342	1610	1692	1.20	0.144
rGO-Elastin	1347	1591	2700	1.16	0.173

**Table 5:** Parameters from FT-IR using an attenuated total reflectance (ATR) for rGO and rGO-proteins.

	C=C (cm^−1^)	Carboxyl C=O (cm^−1^)	Carbonyl C-O (cm^−1^)	H-Bonding (cm^−1^)
rGO	1603	1736	1266	3650
rGO+BSA	1590	1760	1227	3701
rGO+Collagen	1592	1774	1247	3618
rGO+Elastin	1600	1770	1250	3655

**Table 6:** Specific capacitance for the tested hydrogels.

Hydrogels	Specific Capacitance (F/g)
Alginate	6.60 × 10^−6^
rGO + alginate	1.84 × 10^−5^
BSA + rGO + alginate	1.88 × 10^−5^
Collagen + rGO + alginate	3.17 × 10^−5^
elastin + rGO + alginate	1.20 × 10^−5^
